# Supplementary material for: Prevalence, specific and non-specific determinants of complementary medicine use in Switzerland: Data from the 2017 Swiss Health Survey
Source: PLoS One. 2022 Sep 14;17(9):e0274334. doi: 10.1371/journal.pone.0274334 (PMC9473626; doi:10.1371/journal.pone.0274334)
Supplement: S1 Table — (DOCX) [file pone.0274334.s001.docx]

**Table S1.** Sociodemographic and health-related characteristics of responders according to CM category

|  | Traditional Chinese medicine, including acupuncture | | | Homeopathy | |  | Herbal medicine | |  | Other CM therapies | |  |
| --- | --- | --- | --- | --- | --- | --- | --- | --- | --- | --- | --- | --- |
|  | **N=18,557** | |  | **N=18,550** | |  | **N=18,540** | |  | **N=18,523** | |  |
|  | Yes (N=1,349) | No (N=17,208) | p-value | Yes (N=1,731) | No (N=16,819) | p-value | Yes (N=1,369) | No (N=17,171) | p-value | Yes (N=4,314) | No (N=14,209) | p-value |
| **Sociodemographic characteristics** | |  |  |  |  |  |  |  |  |  |  |  |
| Age, years |  |  | p<0.001 |  |  | p<0.001 |  |  | p<0.001 |  |  | p<0.001 |
| 15-24 | 8.1% (6.3-9.8) | 13.0% (12.4-13.6) |  | 14.8% (12.7-16.8) | 12.5% (11.9-13.1) |  | 10.0% (8.0-11.9) | 12.9% (12.3-13.5) |  | 10.4% (9.3-11.5) | 13.3% (12.7-14.0) |  |
| 25-44 | 38.2% (35.0-41.5) | 33.0% (32.1-33.9) |  | 35.2% (32.4-38.0) | 33.2% (32.4-34.1) |  | 35.7% (32.6-38.8) | 33.2% (32.4-34.1) |  | 37.1% (35.3-38.9) | 32.4% (31.4-33.4) |  |
| 45-64 | 37.9% (34.9-41.0) | 33.3% (32.4-34.1) |  | 37.4% (34.6-40.2) | 33.2% (32.4-34.1) |  | 40.1% (37.0-43.1) | 33.1% (32.3-33.9) |  | 37.4% (35.7-39.1) | 32.5% (31.6-33.4) |  |
| 65+ | 15.7% (13.6-17.9) | 20.7% (20.0-21.4) |  | 12.7% (10.9-14.5) | 21.0% (20.4-21.7) |  | 14.3% (12.2-16.4) | 20.8% (20.1-21.5) |  | 15.1% (13.9-16.3) | 21.8% (21.1-22.5) |  |
| Gender, female | 66.6% (63.5-69.7) | 49.2% (48.3-50.1) | p<0.001 | 69.1% (66.4-71.8) | 48.7% (47.8-49.6) | p<0.001 | 71.2% (68.2-74.1) | 48.8% (47.9-49.7) | p<0.001 | 66.3% (64.5-68.0) | 46.0% (45.0-47.0) | p<0.001 |
| Educational level |  |  | p<0.001 |  |  | p<0.001 |  |  | p<0.001 |  |  | p<0.001 |
| Primary education | 12.1% (9.9-14.2) | 17.6% (16.9-18.2) |  | 11.4% (9.7-13.2) | 17.7% (17.0-18.4) |  | 10.6% (8.7-12.4) | 17.6% (17.0-18.3) |  | 10.6% (9.6-11.7) | 19.0% (18.2-19.8) |  |
| Secondary education | 49.0% (45.8-52.2) | 47.6% (46.7-48.5) |  | 45.6% (42.7-48.4) | 47.9% (47.0-48.8) |  | 45.5% (42.3-48.7) | 47.9% (47.0-48.8) |  | 47.1% (45.3-48.8) | 47.9% (46.9-48.8) |  |
| Tertiary education | 38.9% (35.8-42.1) | 34.8% (34.0-35.7) |  | 43.0% (40.2-45.9) | 34.4% (33.6-35.3) |  | 43.9% (40.8-47.1) | 34.5% (33.6-35.4) |  | 42.3% (40.5-44.1) | 33.1% (32.2-34.1) |  |
| Marital status |  |  | p=0.18 |  |  | p<0.001 |  |  | p<0.001 |  |  | p=0.005 |
| Single | 33.4% (30.2-36.6) | 34.2% (33.3-35.1) |  | 37.8% (35.0-40.7) | 33.8% (32.9-34.7) |  | 35.3% (32.2-38.5) | 34.1% (33.2-35.0) |  | 34.2% (32.4-35.9) | 34.2% (33.2-35.2) |  |
| Married | 50.6% (47.3-53.8) | 50.8% (49.9-51.7) |  | 47.6% (44.8-50.5) | 51.1% (50.2-52.0) |  | 47.9% (44.7-51.0) | 51.1% (50.2-52.0) |  | 50.2% (48.4-52.0) | 51.1% (50.1-52.1) |  |
| Divorced/separated | 11.9% (9.8-14.0) | 10.0% (9.5-10.6) |  | 11.6% (9.6-13.6) | 10.0% (9.4-10.6) |  | 12.4% (10.3-14.5) | 9.9% (9.4-10.5) |  | 11.2% (10.1-12.4) | 9.8% (9.2-10.4) |  |
| Widowed | 4.2% (2.9-5.4) | 4.9% (4.5-5.3) |  | 2.9% (1.9-3.9) | 5.0% (4.6-5.4) |  | 4.4% (3.1-5.7) | 4.9% (4.5-5.3) |  | 4.4% (3.7-5.1) | 5.0% (4.5-5.4) |  |
| Housing occupancy status |  |  | p=0.03 |  |  | p<0.001 |  |  | p=0.11 |  |  | p<0.001 |
| Renter | 54.9% (51.7-58.1) | 53.8% (52.9-54.7) |  | 50.9% (48.1-53.8) | 54.2% (53.3-55.1) |  | 51.4% (48.3-54.6) | 54.1% (53.2-54.9) |  | 48.8% (47.0-50.6) | 55.3% (54.3-46.3) |  |
| Owner | 44.5% (41.4-47.7) | 44.7% (43.9-45.6) |  | 47.8% (45.0-50.7) | 44.5% (43.6-45.3) |  | 47.3% (44.2-50.5) | 44.6% (43.7-45.4) |  | 50.2% (48.4-52.0) | 43.2% (42.3-44.2) |  |
| Free housing (paid by employer, relative, friend) | 0.6% (0.2-1.0) | 1.4% (1.2-1.6) |  | 1.3% (0.61-1.92) | 1.4% (1.2-1.6) |  | 1.2% (0.5-2.0) | 1.4% (1.2-1.6) |  | 1.0% (0.7-1.3) | 1.5% (1.2-1.7) |  |
| Occupation |  |  | p=0.002 |  |  | p<0.001 |  |  | p<0.001 |  |  | p<0.001 |
| Economically inactive | 24.2% (21.5-26.9) | 29.1% (28.3-29.9) |  | 23.3% (20.9-25.6) | 29.2% (28.4-30.0) |  | 24.5% (21.8-27.1) | 29.0% (28.2-29.8) |  | 23.4% (21.9-24.8) | 30.2% (29.4-31.1) |  |
| Unemployed/housework | 2.2% (1.1-3.3) | 2.5% (2.2-2.8) |  | 2.2% (1.4-3.1) | 2.5% (2.2-2.8) |  | 2.3% (1.3-3.3) | 2.5% (2.2-2.8) |  | 2.2% (1.6-2.8) | 2.5% (2.2-2.9) |  |
| Employed | 73.6% (70.8-76.4) | 68.4% (67.6-69.3) |  | 74.5% (72.0-77.0) | 68.3% (67.5-69.1) |  | 73.2% (70.5-76.0) | 68.5% (67.7-69.3) |  | 74.5% (72.9-76.0) | 67.2% (66.3-68.1) |  |
| Nationality |  |  | p<0.001 |  |  | p<0.001 |  |  | p<0.001 |  |  | p<0.001 |
| Swiss | 79.5% (76.7-82.3) | 75.5% (74.7-76.3) |  | 83.0% (80.7-85.4) | 75.1% (74.3-76.0) |  | 80.1% (77.3-82.8) | 75.5% (74.6-76.3) |  | 83.1% (81.6-84.6) | 73.8% (72.8-74.7) |  |
| Northern/western European | 8.4% (6.4-10.3) | 8.1% (7.6-8.7) |  | 9.4% (7.4-11.4) | 8.0% (7.5-8.6) |  | 10.1% (7.9-12.2) | 8.0% (7.4-8.6) |  | 8.6% (7.4-9.8) | 8.0% (7.4-8.6) |  |
| South European | 5.7% (4.2-7.3) | 8.0% (7.6-8.5) |  | 4.2% (3.0-5.4) | 8.2% (7.7-8.7) |  | 4.9% (3.6-6.3) | 8.1% (7.6-8.6) |  | 4.3% (3.6-5.0) | 8.9% (8.3-9.4) |  |
| Eastern European | 4.3% (3.0-5.6) | 6.1% (5.6-6.5) |  | 2.7% (1.8-3.6) | 6.2% (5.8-6.7) |  | 4.3% (2.9-5.6) | 6.1% (5.6-6.5) |  | 3.0% (2.3-3.6) | 6.8% (6.3-7.3) |  |
| Non-European | 2.1% (0.7-3.5) | 2.2% (1.9-2.6) |  | 0.6% (0.1-1.1) | 2.4% (2.0-2.7) |  | 0.7% (0.1-1.3) | 2.3% (2.0-2.7) |  | 1.0% (0.6-1.5) | 2.6% (2.2-3.0) |  |
| Linguistic region of Switzerland |  |  | p=0.07 |  |  | p<0.001 |  |  | p<0.001 |  |  | p<0.001 |
| German-speaking incl. Romansh-speaking | 68.5% (65.7-71.3) | 71.4% (70.6-72.1) |  | 63.9% (61.3-66.5) | 71.9% (71.1-72.6) |  | 64.0% (61.1-66.9) | 71.7% (71.0-72.5) |  | 64.4% (62.8-66.1) | 73.1% (72.3-73.9) |  |
| French-speaking | 27.4% (24.7-30.2) | 24.1% (23.4-24.8) |  | 32.1% (29.6-64.6) | 23.6% (22.9-24.3) |  | 32.5% (29.7-35.3) | 23.7% (23.0-24.5) |  | 33.0% (31.4-34.7) | 21.9% (21.1-22.6) |  |
| Italian-speaking | 4.1% (3.2-5.0) | 4.5% (4.2-4.8) |  | 4.0% (3.2-4.8) | 4.5% (4.2-4.8) |  | 3.5% (2.7-4.4) | 4.5% (4.3-4.8) |  | 2.5% (2.1-3.0) | 5.0% (4.7-5.3) |  |
| Region of residence |  |  | p=0.05 |  |  | p<0.001 |  |  | p=0.21 |  |  | p<0.001 |
| Urban region | 66.0% (63.1-69.0) | 62.4% (61.5-63.2) |  | 59.2% (56.4-61.9) | 62.9% (62.1-63.8) |  | 63.7% (60.8-66.7) | 62.5% (61.7-63.4) |  | 59.7% (58.0-61.3) | 63.5% (62.6-64.4) |  |
| Intermediate region | 19.0% (16.5-21.4) | 21.7% (21.0-22.5) |  | 22.6% (20.1-25.0) | 21.4% (20.7-22.2) |  | 19.6% (17.2-22.0) | 21.7% (21.0-22.4) |  | 23.1% (21.6-24.6) | 21.0% (20.3-21.9) |  |
| Rural region | 15.0% (12.9-17.2) | 15.9% (15.3-16.5) |  | 18.3% (16.3-20.3) | 15.6% (15.0-16.2) |  | 16.7% (14.5-18.9) | 15.8% (15.2-16.4) |  | 17.2% (15.9-18.4) | 15.5% (14.8-16.1) |  |
| **Physical health** |  |  |  |  |  |  |  |  |  |  |  |  |
| Body mass index |  |  | p<0.001 |  |  | p<0.001 |  |  | p<0.001 |  |  | p<0.001 |
| Underweight | 4.1% (2.9-5.4) | 3.3% (3.0-3.6) |  | 4.8% (3.6-6.0) | 3.2% (2.9-3.6) |  | 4.1% (2.8-5.3) | 3.3% (3.0-3.6) |  | 4.1% (3.4-4.8) | 3.2% (2.8-3.5) |  |
| Normal | 59.1% (55.9-62.3) | 54.9% (54.0-55.7) |  | 62.5% (59.7-65.2) | 54.5% (53.6-55.4) |  | 61.5% (58.4-64.6) | 54.7% (53.8-55.6) |  | 61.4% (59.7-63.2) | 53.4% (52.4-54.4) |  |
| Overweight | 28.5% (25.6-31.4) | 30.7% (29.9-31.5) |  | 24.6% (22.1-27.1) | 31.1% (30.3-31.9) |  | 26.6% (23.9-29.4) | 30.8% (30.0-31.7) |  | 25.7% (24.2-27.3) | 31.9% (31.0-32.9) |  |
| Obese | 10.9% (6.5-10.1) | 11.1% (10.6-11.7) |  | 8.1% (6.5-9.7) | 11.2% (10.6-11.8) |  | 7.8% (6.1-9.5) | 11.2% (10.6-11.8) |  | 8.8% (7.8-9.8) | 11.5% (10.9-12.2) |  |
| Pregnancy^a^ | 2.9% (1.2-4.6) | 2.8% (2.2-3.4) | 0.67 | 3.5% (1.9-5.0) | 2.7% (2.1-3.3) | p=0.30 | 2.5% (1.0-3.9) | 2.8% (2.3-3.4) | p=0.53 | 2.6% (1.7-3.5) | 2.9% (2.2-3.6) | p=0.62 |
| Physical disorder in the past 4 weeks |  |  | p<0.001 |  |  | p<0.001 |  |  | p<0.001 |  |  | p<0.001 |
| None or few | 30.7% (27.6-33.7) | 45.3% (44.4-46.2) |  | 31.3% (28.5-34.0) | 45.5% (44.6-46.4) |  | 33.3% (30.2-36.4) | 45.1% (44.2-46.1) |  | 33.7% (31.9-35.4) | 47.3% (46.3-48.3) |  |
| Moderate | 39.1% (35.9-42.4) | 33.9% (33.0-34.7) |  | 37.2% (34.3-40.1) | 34.0% (33.1-34.9) |  | 34.2% (31.1-37.3) | 34.3% (33.4-35.2) |  | 36.5% (34.7-38.3) | 33.6% (32.6-34.5) |  |
| Severe | 30.2% (27.2-33.2) | 20.8% (20.1-21.6) |  | 31.5% (28.7-34.3) | 20.6% (19.8-21.3) |  | 32.5% (29.5-35.6) | 20.6% (19.8-21.3) |  | 29.8% (28.1-31.5) | 19.1% (18.3-19.9) |  |
| Sleep disorder |  |  | p<0.001 |  |  | p<0.001 |  |  | p<0.001 |  |  | p<0.001 |
| None or few | 65.0% (61.9-68.1) | 71.5% (70.7-72.3) |  | 68.0% (65.3-70.6) | 71.4% (70.5-72.2) |  | 68.2% (65.3-71.0) | 71.3% (70.5-72.1) |  | 69.5% (67.8-71.1) | 71.6% (70.7-72.5) |  |
| Moderate | 24.7% (21.9-27.4) | 22.5% (21.8-23.3) |  | 23.4% (21.0-25.8) | 22.6% (21.9-23.4) |  | 24.5% (21.9-27.2) | 22.5% (21.8-23.3) |  | 22.6% (21.1-24.1) | 22.7% (21.9-23.5) |  |
| Pathological | 10.4% (8.3-12.4) | 5.9% (5.5-6.4) |  | 8.7% (7.1-10.3) | 6.0% (5.6-6.5) |  | 7.3% (5.7-8.8) | 6.2% (5.7-6.6) |  | 7.9% (7.0-8.9) | 5.8% (5.3-6.2) |  |
| Long-lasting or chronic disease/condition (≥ 6 past months) | 42.8% (39.6-46.0) | 31.4% (30.5-32.2) | p<0.001 | 37.1% (34.3-39.9) | 31.7% (30.9-32.6) | p<0.001 | 36.4% (33.4-39.4) | 31.8% (31.0-32.6) | p<0.001 | 38.3% (36.6-40.1) | 30.5% (29.6-31.4) | p<0.001 |
| Allergies | 29.3% (26.3-32.3) | 24.1% (23.4-24.9) | p<0.001 | 35.0% (32.2-37.8) | 23.5% (22.8-24.3) | p<0.001 | 31.6% (28.7-34.6) | 23.9% (23.2-24.7) | p<0.001 | 29.7% (28.1-31.4) | 23.0% (22.2-23.9) | p<0.001 |
| Cancer | 1.8% (1.1-2.6) | 1.5% (1.3-1.7) | 0.22 | 1.8% (1.1-2.6) | 1.5% (1.3-1.7) | p=0.86 | 1.9% (1.1-2.8) | 1.5% (1.3-1.7) | p=0.18 | 1.4% (1.0-1.9) | 1.6% (1.3-1.8) | p=0.24 |
| Intensity of headache or migraine in the past 4 weeks |  |  | p<0.001 |  |  | p<0.001 |  |  | p<0.001 |  |  | p<0.001 |
| None | 59.6% (56.4-62.8) | 69.8% (69.0-70.7) |  | 60.4% (57.6-63.2) | 69.9% (69.1-70.8) |  | 62.3% (59.2-65.3) | 69.6% (68.8-70.5) |  | 62.4% (60.6-64.1) | 71.0% (70.1-71.9) |  |
| Moderate | 31.1% (28.0-34.1) | 25.1% (24.3-25.8) |  | 31.7% (29.0-34.3) | 24.9% (24.1-25.7) |  | 30.9% (28.0-33.8) | 25.1% (24.3-25.9) |  | 30.6% (29.0-32.3) | 24.0% (23.2-24.9) |  |
| High | 9.3% (7.4-11.3) | 5.1% (4.7-5.5) |  | 8.0% (6.4-9.5) | 5.2% (4.7-5.6) |  | 6.8% (5.2-8.4) | 5.3% (4.9-5.7) |  | 7.0% (6.1-7.9) | 5.05 (4.5-5.4) |  |
| **Mental health** |  |  |  |  |  |  |  |  |  |  |  |  |
| Psychological distress in the past 4 weeks |  |  | p<0.001 |  |  | p<0.001 |  |  | p<0.001 |  |  | p<0.001 |
| Low | 79.6% (77.0-82.3) | 85.9% (85.3-86.5) |  | 81.0% (78.7-83.3) | 85.9% (85.2-86.5) |  | 80.4% (77.9-82.8) | 85.9% (85.2-86.5) |  | 82.2% (80.9-83.6) | 86.4% (85.7-87.1) |  |
| Moderate | 13.7% (11.5-15.9) | 10.5% (9.9-11.0) |  | 12.9% (11.0-14.8) | 10.5% (9.9-11.1) |  | 13.8% (11.7-15.9) | 10.5% (9.9-11.0) |  | 13.0% (11.8-14.2) | 10.0% (9.4-10.6) |  |
| High | 6.7% (5.0-8.4) | 3.6% (3.3-4.0) |  | 6.1% (4.5-7.6) | 3.7% (3.3-4.0) |  | 5.8% (4.3-7.4) | 3.7% (3.3-4.0) |  | 4.8% (4.0-5.6) | 3.6% (3.2-4.0) |  |
| Depression in the past 2 weeks |  |  | p<0.001 |  |  | p<0.001 |  |  | p<0.001 |  |  | p<0.001 |
| None or minimal | 57.8% (54.6-61.1) | 66.0% (65.2-66.9) |  | 57.1% (54.2-60.0) | 66.2% (65.4-67.1) |  | 56.2% (53.0-59.4) | 66.2% (65.3-67.1) |  | 60.6% (58.8-62.4) | 66.9% (65.9-67.8) |  |
| Slight | 30.5% (27.5-33.4) | 25.6% (24.8-26.4) |  | 30.2% (27.5-32.8) | 25.6% (24.8-26.4) |  | 30.6% (27.7-33.6) | 25.6% (24.8-26.4) |  | 28.9% (27.2-30.5) | 25.1% (24.2-26.0) |  |
| Moderate | 7.1% (5.4-8.7) | 5.7% (5.3-6.2) |  | 8.5% (6.8-10.2) | 5.6% (5.2-6.0) |  | 9.6% (7.7-11.6) | 5.5% (5.1-5.9) |  | 7.4% (6.4-8.3) | 5.4% (4.9-5.9) |  |
| Moderately severe | 3.1% (1.9-4.3) | 1.9% (1.6-2.1) |  | 3.2% (2.1-4.2) | 1.8% (1.6-2.1) |  | 2.1% (1.2-2.9) | 1.9% (1.7-2.2) |  | 2.3% (1.8-2.8) | 1.8% (1.6-2.1) |  |
| Severe | 1.5% (0.7-2.4) | 0.8% (0.6-0.9) |  | 1.1% (0.5-1.6) | 0.8% (0.6-1.0) |  | 1.4% (0.7-2.2) | 0.8% (0.6-1.0) |  | 0.9% (0.6-1.2) | 0.8% (0.6-1.0) |  |
| Impact of health concerns on lifestyle |  |  | p<0.001 |  |  | p<0.001 |  |  | p<0.001 |  |  | p<0.001 |
| Living without thinking about health | 8.3% (6.5-10.0) | 12.8% (12.2-13.4) |  | 6.6% (5.2-7.9) | 13.0% (12.4-13.6) |  | 5.6% (4.2-7.0) | 13.0% (12.4-13.6) |  | 7.9% (6.9-8.8) | 13.8% (13.1;14.4) |  |
| Health concerns affect lifestyle | 70.2% (67.2-73.1) | 68.4% (67.6-69.3) |  | 74.8% (72.4-77.3) | 68.0% (67.1-68.8) |  | 74.4% (71.7-77.2) | 68.1% (67.3-68.9) |  | 72.4% (70.8-73.9) | 67.5% (66.5;68.4) |  |
| Health concerns determine lifestyle | 21.5% (18.9-24.2) | 18.8% (18.1-19.5) |  | 18.6% (16.4-20.8) | 19.0% (18.3-19.7) |  | 20.0% (17.522.5) | 18.9% (18.2-19.6) |  | 19.8% (18.4-21.2) | 18.8% (18.0-19.6) |  |
| **Lifestyle** |  |  |  |  |  |  |  |  |  |  |  |  |
| Physical activity |  |  | p=0.34 |  |  | p<0.001 |  |  | p<0.001 |  |  | p<0.001 |
| None | 7.7% (5.9-9.6) | 7.7% (7.2-8.2) |  | 5.1% (3.7-6.4) | 8.0% (7.5-8.5) |  | 5.1% (3.6-6.5) | 7.9% (7.5-8.4) |  | 5.4% (4.6-6.2) | 8.4% (7.8-8.9) |  |
| Partially active | 17.6% (15.1-20.0) | 16.1% (15.5-16.8) |  | 17.2% (15.0-19.3) | 16.2% (15.5-16.8) |  | 16.9% (14.7-19.2) | 16.2% (15.5-16.8) |  | 16.9% (15.6-18.2) | 16.1% (15.4-16.8) |  |
| Sufficiently active | 43.3% (40.1-46.5) | 44.9% (44.0-45.8) |  | 46.8% (44.0-49.7) | 44.6% (43.7-45.5) |  | 46.9% (43.7-50.0) | 44.6% (43.7-45.5) |  | 44.7% (42.9-46.5) | 44.8% (43.8-45.8) |  |
| Trained | 31.4% (28.3-34.4) | 31.3% (30.4-32.1) |  | 30.9% (28.2-33.6) | 31.3% (30.4-32.2) |  | 31.1% (28.1-34.2) | 31.3% (30.4-32.1) |  | 33.0% (31.3-34.8) | 30.8% (29.8-31.7) |  |
| Fruit and/or vegetable consumption |  |  | p<0.001 |  |  | p<0.001 |  |  | p<0.001 |  |  | p<0.001 |
| < 5 days/week | 7.9% (6.0-9.9) | 10.0% (9.5-10.6) |  | 5.1% (3.7-6.4) | 10.3% (9.8-10.9) |  | 4.7% (3.3-6.0) | 10.3% (9.7-10.9) |  | 6.4% (5.5-7.3) | 10.9% (10.3-11.5) |  |
| 0-2 portions/day, ≥5 days/week | 30.3% (27.3-33.3) | 34.9% (34.0-35.7) |  | 26.7% (24.2-29.2) | 35.3% (34.4-36.1) |  | 27.3% (24.5-30.2) | 35.1% (34.2-36.0) |  | 28.8% (27.1-30.4) | 36.1% (35.2-37.1) |  |
| 3-4 portions/day, ≥5 days/week | 37.1% (34.0-40.3) | 33.6% (32.7-34.4) |  | 37.5% (34.7-40.3) | 33.5% (32.6-34.3) |  | 36.9% (33.8-40.0) | 33.6% (32.7-34.4) |  | 36.1% (34.3-37.8) | 33.2% (32.3-34.2) |  |
| ≥5 portions/day, ≥5 days/week | 24.7% (22.0-27.3) | 21.6% (20.8-22.3) |  | 30.7% (28.1-33.4) | 20.9% (20.2-21.7) |  | 31.1% (28.2-34.0) | 21.0% (20.3-21.8) |  | 28.8% (27.2-30.4) | 19.8% (19.0-20.6) |  |
| Daily tobacco consumption |  |  | p=0.06 |  |  | p=0.002 |  |  | p=0.01 |  |  | p<0.001 |
| None | 76.6% (73.9-79.4) | 73.1% (72.3-74.0) |  | 76.0% (73.5-78.5) | 73.1% (72.3-74.0) |  | 76.0% (73.3-78.8) | 73.2% (72.4-74.0) |  | 76.0% (74.5-77.6) | 72.7% (71.8-73.6) |  |
| Occasional smoker | 8.2% (6.4-9.9) | 8.2% (7.7-8.7) |  | 9.0% (7.4-10.7) | 8.1% (7.6-8.6) |  | 8.3% (6.5-10.0) | 8.2% (7.7-8.7) |  | 9.0% (7.9-10.1) | 7.9% (7.4-8.5) |  |
| Daily smoker | 15.2% (12.9-17.6) | 18.7% (17.9-19.4) |  | 14.9% (12.8-17.0) | 18.7% (18.0-19.5) |  | 15.7% (13.3-18.1) | 18.6% (17.9-19.3) |  | 15.0% (16.7-16.3) | 19.4% (18.6-20.2) |  |
| Occasional drunkenness in the past 12 months |  |  | p=0.25 |  |  | p=0.006 |  |  | p=0.50 |  |  | p<0.001 |
| Lifetime non-drinker, abstainer | 19.6% (16.8-22.4) | 16.5% (15.8-17.2) |  | 13.8% (11.7-15.8) | 17.0% (16.3-17.7) |  | 15.1% (12.8-17.4) | 16.8% (16.1-17.5) |  | 13.8% (12.5-15.0) | 17.5% (16.8-18.3) |  |
| None in the past 12 months | 36.6% (33.6-29.7) | 36.1% (35.2-36.9) |  | 36.6% (33.9-39.4) | 36.1% (35.2-36.9) |  | 37.5% (34.5-40.6) | 36.0% (35.1-36.9) |  | 34.7% (33.0-36.4) | 36.5% (35.6-37.5) |  |
| <1/month | 29.9% (27.0-32.8) | 31.7% (30.9-32.5) |  | 34.5% (31.8-37.3) | 31.3% (30.4-32.1) |  | 32.5% (29.5-35.5) | 31.5% (30.7-32.3) |  | 34.7% (32.9-36.4) | 30.7% (29.8-31.6) |  |
| Every month | 10.3% (8.4-12.2) | 12.7% (12.1-13.3) |  | 11.3% (9.6-13.0) | 12.6% (12.0-13.2) |  | 11.9% (9.8-13.9) | 12.6% (12.0-13.2) |  | 13.6% (12.3-14.8) | 12.2% (11.5-12.9) |  |
| ≥ 1/week | 3.6.% (2.3-4.9) | 3.1% (2.8-3.4) |  | 3.8% (2.6-4.9) | 3.1% (2.7-3.4) |  | 3.0% (1.7-4.3) | 3.1% (2.8-3.5) |  | 3.3% (2.6-4.0) | 3.1% (2.7-3.4) |  |
| Last cannabis consumption |  |  | p<0.001 |  |  | p<0.001 |  |  | p<0.001 |  |  | p<0.001 |
| None | 68.0% (64.8-71.2) | 72.0% (71.0-72.9) |  | 66.2% (63.3-69.0) | 72.3% (71.4-73.2) |  | 66.8% (63.7-70.0) | 72.2% (71.3-73.1) |  | 66.9% (65.1-68.7) | 73.2% (72.2-74.2) |  |
| >12 months | 25.9% (22.9-28.9) | 21.1% (20.3-21.9) |  | 24.8% (22.3-27.4) | 21.1% (20.3-21.9) |  | 24.7% (21.9-27.6) | 21.2% (20.4-22.0) |  | 26.0% (24.3-27.7) | 20.1% (19.2-20.9) |  |
| ≤ 12 months | 3.3% (1.9-4.7) | 3.4% (3.1-3.8) |  | 4.4% (3.2-5.7) | 3.3% (2.9-3.7) |  | 3.5% (2.2-4.9) | 3.4% (3.0-3.8) |  | 4.0% (3.2-4.8) | 3.3% (2.9-3.7) |  |
| ≤ 30 days | 2.8% (1.6-3.9) | 3.5% (3.1-3.8) |  | 4.5% (3.2-5.9) | 3.3% (2.9-3.7) |  | 4.9% (3.4-6.4) | 3.3% (2.9-3.6) |  | 3.2% (2.4-3.9) | 3.5% (3.0-3.9) |  |
| **Personal resources and social support** |  |  |  |  |  |  |  |  |  |  |  |  |
| Mastery |  |  | p<0.001 |  |  | p<0.001 |  |  | p<0.001 |  |  | p<0.001 |
| Low | 31.0% (28.0-34.0) | 22.7% (22.0-23.5) |  | 28.6% (25.9-31.2) | 22.8% (22.0-23.6) |  | 31.8% (28.9-34.7) | 22.6% (21.9-23.4) |  | 28.1% (26.5-29.7) | 21.9% (21.1-22.8) |  |
| Moderate | 39.1% (35.8-42.3) | 39.2% (38.4-40.1) |  | 42.1% (39.3-45.0) | 38.9% (38.0-39.8) |  | 41.6% (38.4-44.7) | 39.0% (38.2-39.9) |  | 39.8% (38.0-41.5) | 39.0% (38.1-40.0) |  |
| High | 29.9% (27.0-32.9) | 38.0% (37.2-38.9) |  | 29.3% (26.7-31.9) | 38.3% (37.4-39.2) |  | 26.6% (23.8-29.5) | 38.3% (37.4-39.2) |  | 32.1% (30.4-33.8) | 39.0% (38.1-40.0) |  |
| Social supports |  |  | p=0.06 |  |  | p=0.03 |  |  | p=0.30 |  |  | p<0.001 |
| Low | 9.8% (7.6-11.9) | 9.6% (9.1-10.1) |  | 7.8% (6.1-9.4) | 9.8% (9.2-10.3) |  | 9.6% (7.6-11.6) | 9.6% (9.1-10.2) |  | 7.8% (6.8-8.9) | 10.1% (9.5-10.7) |  |
| Moderate | 42.3% (39.1-45.6) | 45.0% (44.1-46.0) |  | 44.2% (41.3-47.1) | 44.9% (44.0-45.8) |  | 42.7% (39.5-45.9) | 45.0% (44.1-45.9) |  | 42.5% (40.7-44.3) | 45.5% (44.5-46.6) |  |
| High | 47.9% (44.6-51.2) | 45.3% (44.4-46.3) |  | 48.1% (45.2-51.0) | 45.3% (44.4-46.2) |  | 47.7% (44.5-51.0) | 45.4% (44.5-46.3) |  | 49.7% (47.9-51.5) | 44.4% (43.4-45.4) |  |
| **Use of the health care system** | |  |  |  |  |  |  |  |  |  |  |  |
| Consultation with general practitioner in the past 12 months | 81.0% (78.5-83.6) | 69.7% (68.8-70.5) | p<0.001 | 75.3% (72.8-77.8) | 70.0% (69.2-70.9) | p=0.002 | 72.7% (69.8-75.6) | 70.3% (69.4-71.1) | p=0.01 | 74.9% (73.4-76.5) | 69.2% (68.3-70.1) | p<0.001 |
| Consultation with other medical specialists (except gynecologist) in the past 12 months | 59.2% (56.0-62.4) | 41.8% (40.9-42.7) | p<0.001 | 50.2% (47.4-53.1) | 42.4% (41.5-43.3) | p<0.001 | 49.1% (46.0-52.3) | 42.6% (41.7-43.5) | p<0.001 | 52.8% (51.0-54.6) | 40.3% (39.0-41.2) | p<0.001 |
| Supplemental health insurance for complementary medicine |  |  | p<0.001 |  |  | p<0.001 |  |  | p<0.001 |  |  | p<0.001 |
| Yes | 76.6% (73.7-79.5) | 53.4% (52.5-54.3) |  | 74.9% (72.3-77.5) | 53.2% (52.3-54.1) |  | 70.6% (67.5-73.6) | 53.8% (52.9-54.7) |  | 75.6% (74.0-77.2) | 49.2% (48.2-50.2) |  |
| No | 16.6% (14.1-19.2) | 33.0% (32.1-33.9) |  | 15.6% (13.4-17.8) | 33.3% (32.5-34.2) |  | 22.1% (19.4-24.9) | 32.6% (31.7-33.4) |  | 16.5% (15.1-17.9) | 36.2% (35.2-37.1) |  |
| Do not know | 6.8% (5.1-8.5) | 13.6% (13.0-14.3) |  | 9.5% (7.7-11.2) | 13.5% (12.9-14.1) |  | 7.3% (5.5-9.1) | 13.6% (13.0-14.2) |  | 7.9% (6.9-8.9) | 14.6% (13.9-15.3) |  |

Results shown are weighted percentage (95% confidence interval)

Other complementary medicine therapies include shiatsu, reflexology, osteopathy, Ayurveda, naturopathy, kinesiology, Feldenkrais, autogenic training, neural therapy, bioresonance therapy, anthroposophic medicine.

COPD, chronic obstructive pulmonary disease

^a^Among females ≤49 years old: N=4925 in TCM group, N=4923 in homeopathy group, N=4923 in herbal medicine group, N=4923 in other CM therapies group
