# Supplementary material for: Prevalence, specific and non-specific determinants of complementary medicine use in Switzerland: Data from the 2017 Swiss Health Survey
Source: PLoS One. 2022 Sep 14;17(9):e0274334. doi: 10.1371/journal.pone.0274334 (PMC9473626; doi:10.1371/journal.pone.0274334)
Supplement: S2 Table — (DOCX) [file pone.0274334.s002.docx]

**Table S2.** Sociodemographic and health-related characteristics of responders according to CM use and conventional health care use

|  | CM user | | | | General practitioner | | | | Other medical specialists (except gynecologist) | | | | | |
| --- | --- | --- | --- | --- | --- | --- | --- | --- | --- | --- | --- | --- | --- | --- |
|  | **N=18,655** |  |  | **N=18,602** | | |  | **N=18,613** | | | |  | |  |
|  | Yes | No | p-value | Yes | | No | p-value | Yes | | No | p-value | |  |  |
| **Sociodemographic characteristics** |  |  |  |  | |  |  |  | |  |  | |  |  |
| Age, years |  |  | p<0.001 |  | |  | p<0.001 |  | |  | p<0.001 | |  |  |
| 15-24 | 11.0% (10.1-12.0) | 13.2% (12.6-13.9) |  | 12.6% (12.0-13.3) | | 12.5% (11.4-13.6) |  | 10.3% (9.5-11.2) | | 14.3% (13.6-15.1) |  | |  |  |
| 25-44 | 37.0% (35.4-38.5) | 31.8% (30.8-32.8) |  | 29.4% (28.4-30.3) | | 42.7% (41.1-44.4) |  | 28.6% (27.4-29.8) | | 36.9% (35.7-38.0) |  | |  |  |
| 45-64 | 36.7% (35.2-38.2) | 32.3% (31.4-33.2) |  | 33.1% (32.1-34.0) | | 34.7% (33.2-36.2) |  | 35.2% (33.9-36.4) | | 32.3% (31.3-33.4) |  | |  |  |
| 65+ | 15.3% (14.2-16.3) | 22.7% (21.9-23.5) |  | 24.9% (24.1-25.8) | | 10.1% (9.2-11.0) |  | 25.9% (24.8-27.0) | | 16.5% (15.7-17.3) |  | |  |  |
| Gender, female | 64.8% (63.3-66.3) | 44.7% (43.7-45.7) | p<0.001 | 52.0% (51.0-53.0) | | 46.7% (45.1-48.3) | p<0.001 | 52.2% (50.9-53.5) | | 49.1% (48.0-20.3) | p=0.02 | |  |  |
| Educational level |  |  | p<0.001 |  | |  | p<0.001 |  | |  | p<0.001 | |  |  |
| Primary education | 12.0% (11.0-13.0) | 19.6% (18.6-20.2) |  | 18.4% (17.6-19.1) | | 14.5% (13.4-15.6) |  | 15.7% (14.8-16.6) | | 18.4% (17.5-19.3) |  | |  |  |
| Secondary education | 47.1% (45.6-48.7) | 47.9% (46.9-49.0) |  | 49.0% (48.0-50.1) | | 44.6% (42.9-46.2) |  | 48.1% (49.8-49.4) | | 47.4% (46.3-48.6) |  | |  |  |
| Tertiary education | 40.9% (39.4-42.5) | 32.6% (31.7-33.6) |  | 32.6% (31.6-33.6) | | 40.9% (39.3-42.6) |  | 36.2 (35.0-37.5) | | 34.2% (33.1-35.3) |  | |  |  |
| Marital status |  |  | p=0.01 |  | |  | p<0.001 |  | |  | p<0.001 | |  |  |
| Single | 34.5% (33.0-36.1) | 33.8% (32.8-34.8) |  | 32.2% (31.2-33.2) | | 38.2% (36.6-39.9) |  | 30.8% (29.5-32.0) | | 36.5% (35.4-37.7) |  | |  |  |
| Married | 50.1% (48.5-51.7) | 51.2% (50.1-52.2) |  | 51.0% (50.0-52.0) | | 50.7% (49.0-52.3) |  | 51.7% (50.4-53.0) | | 50.1% (49.0-51.3) |  | |  |  |
| Divorced/separated | 11.0% (9.9-12.0) | 9.9% (9.2-10.5) |  | 10.8% (10.1-11.4) | | 8.8% (7.8-9.7) |  | 11.8 (10.9-12.6) | | 9.0% (8.3-9.7) |  | |  |  |
| Widowed | 4.4% (3.8-5.0) | 5.1% (4.7-5.6) |  | 6.0% (5.5-6.5) | | 2.3% (1.9-2.8) |  | 5.7% (5.1-6.3) | | 4.3% (3.9-4.8) |  | |  |  |
| Housing occupancy status |  |  | p<0.001 |  | |  | p<0.001 |  | |  | p=0.14 | |  |  |
| Renter | 50.1% (48.5-51.6) | 55.5% (54.5-56.5) |  | 53.4% (52.4-54.4) | | 55.0% (53.4-56.6) |  | 52.8% (51.5-54.1) | | 54.8% (53.7-55.9) |  | |  |  |
| Owner | 48.8% (47.3-50.4) | 43.0% (42.0-44.0) |  | 45.0% (44.0-46.0) | | 44.1% (42.5-45.7) |  | 45.7% (44.4-47.0) | | 43.9% (42.8-45.1) |  | |  |  |
| Free housing (paid by employer, relative, friend) | 1.1% (0.8-1.4) | 1.5% (1.2-1.7) |  | 1.6% (1.3-1.8) | | 0.9% (0.6-1.2) |  | 1.5% (1.2-1.8) | | 1.3% (1.1-1.5) |  | |  |  |
| Occupation |  |  | p<0.001 |  | |  | p<0.001 |  | |  | p<0.001 | |  |  |
| Economically inactive | 23.8% (22.5-25.1) | 31.0% (30.1-31.9) |  | 33.4% (32.5-34.3) | | 18.2% (17.0-19.3) |  | 34.6% (33.4-35.8) | | 24.6% (23.7-25.6) |  | |  |  |
| Unemployed/housework | 2.2% (1.7-2.7) | 2.5% (2.2-2.9) |  | 2.3% (2.0-2.6) | | 2.8% (2.2-3.4) |  | 2.2% (1.8-2.6) | | 2.6% (2.2-3.0) |  | |  |  |
| Employed | 74.0% (72.7-75.3) | 66.4% (65.5-67.4) |  | 64.3% (63.4-65.3) | | 79.1% (77.8-80.3) |  | 63.2% (62.0-64.4) | | 72.8% (71.8-73.7) |  | |  |  |
| Nationality |  |  | p<0.001 |  | |  | p=0.24 |  | |  | p<0.001 | |  |  |
| Swiss | 81.4% (80.1-82.7) | 73.4% (72.5-74.4) |  | 76.5% (75.5-77.4) | | 74.2% (72.7-75.7) |  | 78.2% (77.0-79.3) | | 74.0% (72.9-75.1) |  | |  |  |
| Northern/western European | 8.7% (7.7-9.7) | 7.9% (7.2-8.6) |  | 7.8% (7.1-8.4) | | 8.9% (7.8-10.0) |  | 8.3% (7.5-9.1) | | 8.0% (7.2-8.7) |  | |  |  |
| South European | 5.1% (4.4-5.8) | 9.1% (8.5-9.7) |  | 7.8% (7.2-8.3) | | 8.3% (7.4-9.2) |  | 7.1% (6.5-7.8) | | 8.6% (7.9-9.2) |  | |  |  |
| Eastern European | 3.4% (2.9-4.0) | 7.0% (6.5-7.5) |  | 6.0% (5.5-6.5) | | 5.8% (5.0-6.5) |  | 4.8% (4.3-5.4) | | 6.8% (6.2-7.4) |  | |  |  |
| Non-European | 1.3% (0.9-1.8) | 2.6% (2.2-3.0) |  | 2.0% (1.6-2.3) | | 2.8% (2.1-3.5) |  | 1.6% (1.2-2.0) | | 2.7% (2.2-3.2) |  | |  |  |
| Linguistic region of Switzerland |  |  | p<0.001 |  | |  | p=0.01 |  | |  | p<0.001 | |  |  |
| German-speaking incl. Romansh-speaking | 64.8% (63.3-66.2) | 73.8% (72.9-74.6) |  | 71.0% (70.1-71.9) | | 71.5% (70.2-72.9) |  | 69.9% (68.8-71.0) | | 72.1% (71.1-73.0) |  | |  |  |
| French-speaking | 31.7% (30.4-33.1) | 21.4% (20.6-22.2) |  | 24.5% (23.7-25.3) | | 24.2% (22.9-25.5) |  | 25.8% (24.8-26.9) | | 23.3% (22.4-24.2) |  | |  |  |
| Italian-speaking | 3.5% (3.1-3.9) | 4.8% (4.5-5.2) |  | 4.5% (4.2-4.8) | | 4.3% (3.8-4.8) |  | 4.2% (3.8-4.6) | | 4.6% (4.3-5.0) |  | |  |  |
| Region of residence |  |  | p<0.001 |  | |  | p=0.51 |  | |  | p<0.001 | |  |  |
| Urban region | 61.0% (59.5-62.5) | 63.4% (62.4-64.3) |  | 62.8% (61.9-63.8) | | 62.2% (60.6-63.8) |  | 64.1% (62.9-65.3) | | 61.6% (60.5-62.7) |  | |  |  |
| Intermediate region | 22.1% (20.9-23.4) | 21.2% (20.4-22.1) |  | 21.4% (20.6-22.2) | | 21.8% (20.5-23.2) |  | 20.9% (19.9-22.0) | | 21.9% (21.0-22.9) |  | |  |  |
| Rural region | 16.9% (15.8-18.0) | 15.4% (14.7-16.1) |  | 15.8% (15.1-16.5) | | 16.0% (15.9-17.1) |  | 15.0% (14.1;15.8) | | 16.5% (15.7;17.3) |  | |  |  |
| **Physical health** |  |  |  |  | |  |  |  | |  |  | |  |  |
| Body mass index |  |  | p<0.001 |  | |  | p<0.001 |  | |  | p<0.001 | |  |  |
| Underweight | 4.0% (3.4-4.7) | 3.1% (2.7-3.4) |  | 3.3% (2.9-3.6) | | 3.6% (3.1-4.2) |  | 3.3% (2.8-3.8) | | 3.4% (3.0-3.8) |  | |  |  |
| Normal | 61.1% (59.6-62.6) | 52.7% (51.6-53.7) |  | 52.8% (51.8-53.8) | | 60.6% (59.0-62.2) |  | 52.3% (51.0-53.6) | | 57.2% (56.1-58.4) |  | |  |  |
| Overweight | 26.2% (24.8-27.6) | 32.4% (31.4-33.3) |  | 31.6% (30.7-32.5) | | 28.1% (26.6-30.0) |  | 31.4% (30.2-32.6) | | 29.9% (28.8-31.0) |  | |  |  |
| Obese | 8.7% (7.8-9.6) | 11.9% (11.2-12.6) |  | 12.3% (11.7-13.0) | | 7.7% (6.8-8.6) |  | 13.0% (12.1-13.9) | | 9.5% (8.8-10.1) |  | |  |  |
| Pregnancy^a^ | 2.9% (2.1-3.7) | 2.7% (2.0-3.4) | p=0.66 | 2.7% (2.1-3.4) | | 3.0% (2.0-4.0) | p=0.15 | 1.9% (1.3-2.6) | | 3.3% (2.5-4.1) | p=0.09 | |  |  |
| Physical disorder in the past 4 weeks |  |  | p<0.001 |  | |  | p<0.001 |  | |  | p<0.001 | |  |  |
| None or few | 34.5% (32.9-36.0) | 48.1% (47.1-49.2) |  | 39.5% (38.5-40.5) | | 55.5% (53.9-57.2) |  | 36.2% (24.9-37.5) | | 50.3% (49.1-51.5) |  | |  |  |
| Moderate | 36.2% (34.7-37.8) | 33.4% (32.4-34.4) |  | 35.3% (343-36.3) | | 31.6% (30.1-33.1) |  | 35.5% (34.2-36.8) | | 33.3% (32.2-34.4) |  | |  |  |
| Severe | 29.3% (27.9-30.8) | 18.4% (17.6-19.3) |  | 25.2% (24.3-26.1) | | 12.9% (11.8-14.0) |  | 28.3 (27.1-29.5) | | 16.4% (15.5-17.3) |  | |  |  |
| Sleep disorder |  |  | p<0.001 |  | |  | p<0.001 |  | |  | p<0.001 | |  |  |
| None or few | 68.5% (67.1-70.0) | 71.9% (71.0-72.8) |  | 68.5% (67.5-69.4) | | 77.0% (75.6-78.3) |  | 66.3% (65.1-67.6) | | 74.5% (73.5-75.5) |  | |  |  |
| Moderate | 23.4% (22.1-24.7) | 22.6% (21.7-23.4) |  | 24.4% (23.6-25.3) | | 18.8% (17.5-20.0) |  | 25.1% (24.0-26.2) | | 21.1% (20.1-22.0) |  | |  |  |
| Pathological | 8.1% (7.3-9.0) | 5.5% (5.0-6.0) |  | 7.1% (6.6-7.6) | | 4.3% (3.6-5.0) |  | 8.5% (7.8-9.3) | | 4.5% (3.9-5.0) |  | |  |  |
| Long-lasting or chronic disease/condition (≥ 6 past months) | 37.9% (36.4-39.4) | 30.0% (29.1-30.9) | p<0.001 | 39.0% (38.0-40.0) | | 16.0% (14.8-17.1) | p<0.001 | 46.2% (44.9-47.5) | | 21.6% (20.7-22.6) | p<0.001 | |  |  |
| Allergies | 29.7% (28.2-31.1) | 22.4% (24.5-23.3) | p<0.001 | 25.4% (24.5-26.3) | | 22.3% (20.9-23.6) | p<0.001 | 27.1% (26.0-28.3) | | 22.4% (21.4-23.4) | p<0.001 | |  |  |
| Cancer | 1.6% (1.3-2.0) | 1.5% (1.3-1.7) | p=0.95 | 1.9% (1.7-2.2) | | 0.6% (0.4-0.9) | p<0.001 | 3.0% (2.6-3.5) | | 0.4% (0.3-0.5) | p<0.001 | |  |  |
| Intensity of headache or migraine in the past 4 weeks |  |  | p<0.001 |  | |  | p<0.001 |  | |  | p<0.001 | |  |  |
| None | 62.6% (61.1-64.1) | 71.8% (70.8-72.7) |  | 68.2% (67.2-69.1) | | 71.6% (70.1-73.1) |  | 67.7% (66.5-68.9) | | 70.3% (69.2-71.3) |  | |  |  |
| Moderate | 30.2% (28.8-31.7) | 23.5% (22.6-24.4) |  | 25.7% (24.8-26.6) | | 24.8% (23.4-26.3) |  | 25.3 (24.2-26.4) | | 25.5% (24.5-26.5) |  | |  |  |
| High | 7.2% (6.4-8.0) | 4.7% (4.2-5.2) |  | 6.1% (5.6-6.6) | | 3.5% (2.9-4.1) |  | 7.0% (6.3-7.7) | | 4.2% (3.7-4.7) |  | |  |  |
| **Mental health** |  |  |  |  | |  |  |  | |  |  | |  |  |
| Psychological distress in the past 4 weeks |  |  | p<0.001 |  | |  | p<0.001 |  | |  | p<0.001 | |  |  |
| Low | 82.0% (80.7-83.2) | 86.8% (86.1-87.5) |  | 83.5% (82.8-84.3) | | 90.0% (89.0-91.0) |  | 81.0% (80.0-82.0) | | 88.8% (88.0-89.5) |  | |  |  |
| Moderate | 13.2% (12.1-14.2) | 9.7 (9.1-10.4) |  | 11.8% (11.2-12.5) | | 8.0% (7.1-8.9) |  | 12.9% (12.0-13.8) | | 9.0% (8.3-9.5) |  | |  |  |
| High | 4.9% (4.2-5.6) | 3.5% (3.1-3.9) |  | 4.7% (4.2-5.1) | | 2.0% (1.5-2.5) |  | 6.1% (5.5-6.7) | | 2.2% (1.9-2.6) |  | |  |  |
| Depression in the past 2 weeks |  |  | p<0.001 |  | |  | p<0.001 |  | |  | p<0.001 | |  |  |
| None or minimal | 59.8% (58.2-61.3) | 67.8% (66.8-68.8) |  | 62.4% (61.4-63.4) | | 72.9% (71.4-74.3) |  | 60.2% (58.9-61.5) | | 69.4% (68.3-70.5) |  | |  |  |
| Slight | 29.3% (27.9-30.7) | 24.6% (23.7-25.5) |  | 27.7% (26.8-28.7) | | 21.6% (20.3-23.0) |  | 28.6% (27.4-29.8) | | 24.0% (23.0-25.0) |  | |  |  |
| Moderate | 7.5% (6.6-8.4) | 5.1% (4.7-5.6) |  | 6.6% (6.1-7.1) | | 4.0% (3.4-4.7) |  | 7.5% (6.8-8.2) | | 4.6% (4.1-5.0) |  | |  |  |
| Moderately severe | 2.4% (1.9-2.9) | 1.7% (1.4-2.0) |  | 2.3% (2.0-2.6) | | 1.0% (0.7-1.3) |  | 2.4% (2.0-2.8) | | 1.6% (1.3-1.9) |  | |  |  |
| Severe | 1.0% (0.7-1.3) | 0.8% (0.5-1.0) |  | 1.0% (0.8-1.2) | | 0.5% (0.2-0.8) |  | 1.3% (1.0-1.6) | | 0.4% (0.3-0.6) |  | |  |  |
| Impact of health concerns on lifestyle |  |  | p<0.001 |  | |  | p<0.001 |  | |  | p<0.001 | |  |  |
| Living without thinking about health | 8.2% (7.3-9.0) | 14.2% (13.5-14.9) |  | 11.4% (10.8-12.1) | | 14.9% (13.8-16.1) |  | 10.1% (9.4-10.9) | | 14.2% (13.4-15.0) |  | |  |  |
| Health concerns affect lifestyle | 72.4% (71.0-73.7) | 66.8% (65.9-67.8) |  | 68.5% (67.5-69.4) | | 68.6% (67.1-70.1) |  | 69.9% (68.7-71.1) | | 67.3% (66.3-68.4) |  | |  |  |
| Health concerns determine lifestyle | 19.5% (18.2-20.7) | 19.0% (18.1-19.8) |  | 20.1% (19.3-21.0) | | 16.5% (15.2-17.7) |  | 19.9% (18.9-21.0) | | 18.4% (17.5-19.3) |  | |  |  |
| **Lifestyle** |  |  |  |  | |  |  |  | |  |  | |  |  |
| Physical activity |  |  | p<0.001 |  | |  | p<0.001 |  | |  | p<0.001 | |  |  |
| None | 5.8% (5.0-6.5) | 8.6% (8.1-9.2) |  | 8.6% (8.0-9.1) | | 5.8% (5.0-6.6) |  | 9.0% (8.2-9.7) | | 6.9% (6.3-7.5) |  | |  |  |
| Partially active | 16.7% (15.6-17.9) | 16.0% (15.3-16.8) |  | 16.2% (15.5-16.9) | | 16.3% (15.2-17.5) |  | 15.8% (14.9-16.8) | | 16.5% (15.7-17.4) |  | |  |  |
| Sufficiently active | 44.5% (42.9-46.0) | 44.8% (43.8-45.8) |  | 44.8% (43.8-45.8) | | 44.6% (43.0-46.2) |  | 44.5% (43.2-45.8) | | 44.9% (43.7-46.0) |  | |  |  |
| Trained | 33.0% (31.5-34.5) | 30.5% (29.6-31.5) |  | 30.5% (29.5-31.4) | | 33.2% (31.7-34.8) |  | 30.7% (29.4-31.9) | | 31.7% (30.6-32.8) |  | |  |  |
| Fruit and/or vegetable consumption |  |  | p<0.001 |  | |  | p=0.90 |  | |  | p<0.001 | |  |  |
| < 5 days/week | 6.6% (5.7-7.4) | 11.2% (10.6-11.9) |  | 9.9% (9.3-10.5) | | 9.7% (8.7-10.7) |  | 8.6% (7.9-9.3) | | 10.8% (10.1-11.6) |  | |  |  |
| 0-2 portions/day, ≥5 days/week | 29.9% (28.5-31.3) | 36.4% (35.4-37.4) |  | 34.9% (33.9-35.8) | | 33.8% (32.2-35.3) |  | 34.1% (32.9-35.3) | | 34.9% (33.8-36.0) |  | |  |  |
| 3-4 portions/day, ≥5 days/week | 36.3% (34.7-37.8) | 32.8% (31.8-33.8) |  | 33.6% (32.7-34.6) | | 34.2% (32.7-35.8) |  | 34.9% (33.6-36.1) | | 33.0% (32.0-34.1) |  | |  |  |
| ≥5 portions/day, ≥5 days/week | 27.3% (25.9-28.7) | 19.5% (18.7-20.4) |  | 21.6% (20.8-22.4) | | 22.3% (20.9-23.6) |  | 22.4% (21.4-23.5) | | 21.3% (20.3-22.2) |  | |  |  |
| Daily tobacco consumption |  |  | p<0.001 |  | |  | p=0.004 |  | |  | p=0.82 | |  |  |
| None | 75.9% (74.5-77.3) | 72.4% (71.4-73.3) |  | 73.9% (73.0-74.8) | | 72.3% (70.8-73.8) |  | 73.6% (72.5-74.8) | | 73.2% (72.1-74.2) |  | |  |  |
| Occasional smoker | 9.0% (8.1-9.9) | 7.8% (7.3-8.4) |  | 7.7% (7.1-8.3) | | 9.3% (8.3-10.3) |  | 8.4% (7.6-9.1) | | 8.0% (7.4-8.7) |  | |  |  |
| Daily smoker | 15.1% (14.0-16.2) | 19.8% (19.0-20.6) |  | 18.4% (17.6-19.2) | | 18.4% (17.1-19.7) |  | 18.0% (17.0-19.0) | | 18.8% (17.9-19.7) |  | |  |  |
| Occasional drunkenness in the past 12 months |  |  | p<0.001 |  | |  | p<0.001 |  | |  | p<0.001 | |  |  |
| Lifetime non-drinker, abstainer | 14.7% (13.5-15.8) | 17.7% (16.9-18.5) |  | 17.7% (17.0-18.5) | | 14.5% (13.3-15.6) |  | 16.2% (15.3-17.2) | | 17.2% (16.3-18.1) |  | |  |  |
| None in the past 12 months | 35.1% (33.6-36.6) | 36.6% (35.6-37.6) |  | 37.4% (36.5-38.4) | | 33.0% (31.5-34.6) |  | 37.7% (36.4-38.9) | | 34.9% (33.9-36.0) |  | |  |  |
| <1/month | 34.1% (32.6-35.6) | 30.4% (29.4-31.3) |  | 30.5% (29.6-31.5) | | 33.8% (32.2-35.3) |  | 31.1% (29.9-32.4) | | 31.7% (30.7-32.8) |  | |  |  |
| Every month | 12.9% (11.8-14.0) | 12.3% (11.6-13.0) |  | 11.4% (10.7-12.0) | | 15.1% (13.9-16.4) |  | 11.8% (11.0-12.7) | | 12.9% (12.1-13.7) |  | |  |  |
| ≥ 1/week | 3.2% (2.6-3.8) | 3.1% (2.7-3.5) |  | 2.9% (2.6-3.3) | | 3.6% (3.0-4.2) |  | 3.1% (2.6-3.5) | | 3.2% (2.7-3.6) |  | |  |  |
| Last cannabis consumption |  |  | p<0.001 |  | |  | p<0.001 |  | |  | p=0.003 | |  |  |
| None | 66.9% (65.3-68.5) | 73.9% (72.9-74.9) |  | 72.7% (71.7-73.7) | | 69.7% (68.2-71.3) |  | 70.2% (68.9-71.5) | | 73.0% (71.9-74.1) |  | |  |  |
| >12 months | 25.5% (24.0-27.0) | 19.6% (18.7-20.5) |  | 20.5% (19.6-21.4) | | 23.3% (21.9-24.8) |  | 22.3% (21.1-23.5) | | 20.7% (19.7-21.7) |  | |  |  |
| ≤ 12 months | 4.0% (3.3-4.6) | 3.2% (2.8-3.6) |  | 3.5% (3.0-3.9) | | 3.3% (2.7-3.9) |  | 3.7% (3.1-4.2) | | 3.2% (2.7-3.7) |  | |  |  |
| ≤ 30 days | 3.7% (3.0-4.3) | 3.3% (2.9-3.7) |  | 3.3% (2.9-3.7) | | 3.6% (2.9-4.4) |  | 3.8% (3.2-4.5) | | 3.1% (2.6-3.6) |  | |  |  |
| **Personal resources and social support** |  |  |  |  | |  |  |  | |  |  | |  |  |
| Mastery |  |  | p<0.001 |  | |  | p<0.001 |  | |  | p<0.001 | |  |  |
| Low | 28.0% (26.6-29.4) | 21.4% (20.5-22.3) |  | 25.4% (24.5-26.3) | | 18.4% (17.1-19.6) |  | 27.5% (26.3-28.6) | | 20.2% (19.2-21.1) |  | |  |  |
| Moderate | 40.6% (39.042.1) | 38.7% (37.7-39.7) |  | 39.1% (38.1-40.1) | | 39.5% (37.9-41.1) |  | 39.1% (27.8-40.4) | | 39.3% (38.2-40.5) |  | |  |  |
| High | 31.4% (30.0-32.9) | 39.9% (28.9-41.0) |  | 35.5% (34.5-36.5) | | 42.2% (10.6-43.8) |  | 33.4% (32.2-34.7) | | 40.5% (39.4-41.7) |  | |  |  |
| Social supports |  |  | p<0.001 |  | |  | p<0.001 |  | |  | p<0.001 | |  |  |
| Low | 8.4% (7.5-9.3) | 10.2% (9.6-10.8) |  | 10.6% (10.0-11.2) | | 7.5% (6.6-8.3) |  | 11.1% (10.3-12.0) | | 8.5% (7.9-9.2) |  | |  |  |
| Moderate | 43.3% (41.7-44.9) | 45.5% (44.5-46.7) |  | 44.9% (43.9-46.0) | | 44.5% (42.9-46.2) |  | 45.2% (43.9-46.6) | | 44.6% (43.4-45.8) |  | |  |  |
| High | 48.3% (46.7-49.9) | 44.3% (43.3-45.3) |  | 44.5% (43.4-45.5) | | 48.0% (46.4-49.7) |  | 43.6% (42.3-44.9) | | 46.9% (45.7-48.1) |  | |  |  |
| **Use of the health care system** |  |  |  |  | |  |  |  | |  |  | |  |  |
| Consultation with general practitioner in the past 12 months | 75.1% (73.7-76.5) | 68.7% (67.7-69.7) | p<0.001 | - | | - | - | 85.0% (84.0-85.9) | | 59.6% (58.5-60.8) | p<0.001 | |  |  |
| Consultation with other medical specialists (except gynecologist) in the past 12 months | 51.9% (50.4-53.5) | 39.5% (38.5-40.5) | p<0.001 | 51.9% (50.9-52.9) | | 22.0% (20.7-23.3) | p<0.001 | - | | - | - | |  |  |
| Supplemental health insurance for complementary medicine |  |  | p<0.001 |  | |  | p<0.001 |  | |  | p<0.001 | |  |  |
| Yes | 72.7% (71.3-74.1) | 47.8% (46.8-48.8) |  | 56.2% (55.2-57.2) | | 52.2% (50.6-53.8) |  | 59.2% (58.0-60.5) | | 51.9% (50.7-53.1) |  | |  |  |
| No | 18.7% (17.4-19.9) | 37.2% (36.2-38.2) |  | 30.6% (29.6-31.6) | | 34.8% (33.3-36.4) |  | 29.0% (27.8-30.2) | | 33.9% (32.8-35.0) |  | |  |  |
| Do not know | 8.6% (7.7-9.5) | 15.0% (14.2-15.7) |  | 13.2% (12.5-13.9) | | 12.9% (11.8-14.1) |  | 11.7% (10.9-12.6) | | 14.2% (13.4-15.0) |  | |  |  |

Results shown are weighted percentage (95% confidence interval)

CM user includes users of TCM including acupuncture, homeopathy, herbal medicine, shiatsu, reflexology, osteopathy, Ayurveda, naturopathy, kinesiology, Feldenkrais, autogenic training, neural therapy, bioresonance therapy and anthroposophic medicine.

^a^Among females ≤49 years old: N=4934 in complementary medicine group, N=4916 in general practitioner group, N=4922 in specialist group
